# The Effects of Exercise on Indirect Markers of Gut Damage and Permeability: A Systematic Review and Meta-analysis

**DOI:** 10.1007/s40279-020-01348-y

**Published:** 2020-11-17

**Authors:** Sarah Chantler, Alex Griffiths, Jamie Matu, Glen Davison, Ben Jones, Kevin Deighton

**Affiliations:** 1 Carnegie Applied Rugby Research (CARR) Centre, Institute for Sport, Physical Activity and Leisure, Leeds Beckett University, Cavendish G08, Headingley Campus, Leeds, LS6 3QT UK; 2 Yorkshire Carnegie Rugby Union Club, Leeds, UK; 3 School of Clinical and Applied Sciences, Leeds Beckett University, Leeds, UK; 4 Endurance Research Group, School of Sport and Exercise Sciences, University of Kent, Canterbury, UK; 5 School of Science and Technology, University of New England, Armidale, NSW Australia; 6 Division of Exercise Science and Sports Medicine, Department of Human Biology, Faculty of Health Sciences, The University of Cape Town and the Sports Science Institute of South Africa, Cape Town, South Africa; 7 Leeds Rhinos Rugby League Club, Leeds, UK; 8 England Performance Unit, Rugby Football League, Leeds, UK

## Abstract

**Aim:**

Exercise appears to cause damage to the endothelial lining of the human gastrointestinal tract and elicit a significant increase in gut permeability.

**Objective:**

The aim of this review was to determine the effect of an acute bout of exercise on gut damage and permeability outcomes in healthy populations using a meta-analysis.

**Methods:**

PubMed, The Cochrane Library as well as MEDLINE, SPORTDiscus and CINHAL, via EBSCOhost were searched through February 2019. Studies were selected that evaluated urinary (ratio of disaccharide/monosaccharide excretion) or plasma markers [intestinal Fatty Acid Binding Protein (i-FABP)] of gut permeability and gut cell damage in response to a single bout of exercise.

**Results:**

A total of 34 studies were included. A random-effects meta-analysis was performed, and showed a large and moderate effect size for markers of gut damage (i-FABP) (ES 0.81; 95% CI 0.63–0.98; *n* = 26; *p* < 0.001) and gut permeability (Disaccharide Sugar/Monosaccharide Sugar) (ES 0.70; 95% CI 0.29–1.11; *n* = 17; *p* < 0.001), respectively. Exercise performed in hot conditions (> 23 °C) further increased markers of gut damage compared with thermoneutral conditions [ES 1.06 (95% CI 0.88–1.23) vs. 0.66 (95% CI 0.43–0.89); *p* < 0.001]. Exercise duration did not have any significant effect on gut damage or permeability outcomes.

**Conclusions:**

These findings demonstrate that a single bout of exercise increases gut damage and gut permeability in healthy participants, with gut damage being exacerbated in hot environments. Further investigation into nutritional strategies to minimise gut damage and permeability after exercise is required. PROSPERO database number (CRD42018086339).

**Electronic supplementary material:**

The online version of this article (10.1007/s40279-020-01348-y) contains supplementary material, which is available to authorized users.

## Key Points

**Table:** 

This meta-analysis of 34 studies confirms that a single bout of exercise results in damage of the endothelial lining and increases permeability of the gut
Duration of exercise was not a significant contributor to gut damage or increased gut permeability
Hot environments are likely to exacerbate the impact of exercise. This is due to the increased redistribution of blood flow to the skin for thermoregulation and increased hypoxia at the level of the gut lining

## Introduction

Increased gut permeability and gut endothelial damage have been observed in a variety of gastrointestinal and metabolic disorders [1, 2]. Similarly, exercise has been shown to cause increased gut damage and gut permeability in healthy participants [3]. The evidence suggests that in response to exercise, sympathetic nervous system activation causes splanchnic hypoperfusion and hypoxia as blood flow to the skeletal muscle, heart and lungs is prioritised. Decreased gut perfusion contributes to epithelial injury and alterations in endothelial tight junctions [3]. Decreased clearing of metabolites as a result of the hypoxia has also been cited as a contributor to cell injury [3]. Further damage may come as a result of reperfusion post-exercise, which consequently increases gut permeability. Increased gut permeability may enable the translocation of pathogens into the blood stream, in turn triggering pro-inflammatory immune responses. It is hypothesised that this cascade may increase gut symptoms, impair nutrient absorption and possibly increase in the risk of illness, with some preliminary evidence in support this [4, 5], all of which contribute negatively to wellbeing. At this point, robust evidence to support the full hypothesis is currently lacking.

Typically, research investigating exercise-associated gut damage and permeability has focussed on healthy individuals using endurance-style protocols [6–9]. Endothelial cell damage can be approximated by measuring the change in plasma intestinal fatty acid binding protein (i-FABP) levels. This transporter protein is found on the upper luminal surface of the endothelial cell and any plasma increases reflect a breakdown in endothelial cell integrity. It has been proposed as a marker of early gut damage in intestinal injury [10], and has been shown to increase significantly in response to both anti-inflammatories, splanchnic hypoxia, oxidative stress, hyperthermia and mechanical stress related to exercise [11, 12]. Markers assessing an increase in intestinal permeability reflect a deterioration of the tight junctions between the endothelial cells. This can be measured via the urinary ratio of ingested non-digestible sugars (disaccharide and monosaccharide) or other similar non-digestible molecules (e.g., iohexol) that cross the gut lining [1]. Different sugars may illustrate permeability in different parts of the intestine due to their individual properties [13, 14] and there are a variety of protocols for urine collection time [15, 16]. Other markers such as polyethylene glycols and radioactive labelled Cr-EDTA are less common in studies with healthy populations [17]. Bacterial translocation and inflammation have also been estimated using various blood markers in both clinical diagnosis and healthy populations [1].

Studies have shown that significant shunting of blood away from the gut occurs within the first ten minutes of exercise [12]. Intestinal permeability increases significantly once the exercise stimulus is above eighty percent of maximal oxygen uptake [18]. Increased permeability has also been found post marathon (~ 215 min), despite the lower intensity of long distance running [7]. This may highlight the role of duration as well as intensity as contributing factors towards the thermal, oxidative, and mechanical stressors. A hot environment may exacerbate both gut damage and permeability via increases in thermal stress in conjunction with gut hypoxia [19]. However, results are conflicting as to whether any of these factors (heat, intensity, duration) take a dominant lead in increasing the risk of gut damage [6, 20, 21]. Unfortunately, there are few studies that have had the capacity to examine the relative importance and contribution of each of these factors, and their interaction in either laboratory or race (more ecologically valid) settings. With the high occurrence of gut symptoms reported by endurance athletes during races and events [22], further understanding of exercise itself as a contributor to gut discomfort and damage may be useful for athlete management.

Considering the recent interest in gut health and the impact of exercise, a systematic evaluation of the literature is required to determine the magnitude of these effects, as well as the potential influence of factors such as exercise intensity, duration and environmental conditions. Thus, the purpose of this research was to provide a systematic review and meta-analysis of studies that assessed the gut damage and permeability responses to an acute bout of exercise. Meta-regression analysis was also performed to identify exercise and environmental characteristics associated with the magnitude of gut damage and permeability responses to exercise. Understanding these effects provides a basis for identifying athletes at risk of gastrointestinal damage to enable interventions to optimise health and performance.

## Methods

This systematic review and meta-analysis was prospectively registered with the PROSPERO database (CRD42018086339) and was completed in accordance with PRISMA (Preferred Reporting Items for Systematic Review and Meta-analysis) guidelines [23].

### Literature Search

Pubmed and the Cochrane Library, as well as MEDLINE, CINAHL and SPORTDiscus via EBSCOhost were searched through to the 28th of February 2019. Keywords searches were performed for ‘gut’, ‘gastrointestinal’, ‘GI’, ‘intestines’, ‘intestinal’, ‘mucosal’, ‘splanchnic’, ‘permeability’, ‘leaky’, ‘hyperpermeability’, ‘function’, ‘dysfunction’, ‘injury’, ‘exercise’, ‘training’, ‘endurance’, ‘physical activity’. Reference lists of eligible studies and review articles were also searched. Publication date and language restrictions were not applied. As a means to minimise potential publication bias, grey literature was included in search results. Details of the specific search strategy for each database can be found in supplementary material.

### Inclusion Criteria

Studies were included if the participants were healthy, 18–65 years of age, without any history of gastrointestinal illness or any other inflammatory, metabolic, cardiovascular, neurological or psychological disease(s). These criteria were selected to target participants free of any disease or age related outcomes that may confound the response to exercise. Studies were required to include a urinary- or plasma-based measure of gastrointestinal damage or permeability (e.g., urinary lactulose/rhamnose ratio or plasma intestinal fatty-acid binding protein) or a plasma maker of bacterial translocation (e.g., lipopolysaccharide). All studies were required to have either an equivalent resting control trial or a resting pre-exercise data collection point, as well as an exercise/post exercise value, using a within-subjects design. If data were collected during exercise, then only the measure directly after the completion of exercise was extracted. Studies were limited to a single acute bout of exercise to limit the effect of training or cumulative damage. No restrictions were placed on the training status of the participants. If there was a specific nutritional intervention, only data for the placebo trial were extracted. For studies that examined the influence of hydration, all data were extracted.

Two researchers (SC and AG) independently assessed studies for inclusion and later compared notes to reach a mutual consensus. Disagreements about the eligibility of any particular studies were resolved by a third reviewer (KD). Potential studies that could be included based on their title or abstract were retrieved in full-text and reviewed against the inclusion/exclusion criteria independently by two researchers (SC and AG) with a third researcher (KD) used to settle any disputes. In total 34 studies met the inclusion criteria and were included in the meta-analysis (Supplementary Table 1).

### Data Extraction

Data were extracted independently by two researchers (SC and AG) into a standardised spreadsheet, which included (i) characteristics of articles valid for review; (ii) the Cochrane Collaboration's tool for assessing risk of bias and (iii) outcome data suitable for successive analysis based on mean, standard deviation (SD) and sample size. Additional data were collected for study design; participant characteristics; the mode, volume and intensity of exercise; reporting of gastrointestinal symptoms (GIS), dietary methods prior to trial and gut permeability assessment methods. Where a study provided data for more than one environmental or exercise condition, all data were extracted. Where values were only presented in figure form, the figure was digitized using graph digitizer software (DigitizeIt, Germany) and the means and SD/Standard Error of the Mean (SEM) were manually measured at the pixel level to the scale provided on the figure. If individual data points were reported graphically, all points were plotted and the mean and SD were calculated. If multiple time points were reported for i-FABP post exercise, the time point closest to the end of exercise was used as the post-exercise measure. If certain forms of data were not presented, the authors were contacted for the raw data.

### Assessment of Risk of Bias in Included Studies

To assess the risk of bias for included studies, the Cochrane Collaboration tool for assessing bias was used independently by two reviewers (SC and AG) [24]. Each study was assessed using the domains of sequence generation, allocation concealment, blinding of participants, personnel and outcome assessors, incomplete outcome data, selective reporting and other sources of bias. Each criterion was adjudged as ‘low risk’ or ‘high risk’ by the researchers. If the judgement was unclear due to insufficient detail, an ‘unclear’ risk was given [24]. Disagreements were solved initially via discussion between the two independent reviewers; however, a third reviewer (KD) was consulted for any necessary dispute resolution.

### Statistical Analysis

Data for the meta-analysis were transformed into mean and standard deviations (SD) if required. Revised methods from Wan et al. were used when the median and Inter quartile range (IQR) were reported to transform the data into mean and SD [25]. If the standard error was reported, this was transformed into an SD via the standard equation [26]. Hedges’ *g* and 95% confidence intervals (CI) were calculated using Comprehensive Meta-analysis (CMA) software (version 3, Biostat, Englewood, NJ, USA). A random-effects meta-analysis was performed by SC, AG and KD. A random effects model was employed for all analyses based on the assumption that heterogeneity would exist between included studies due to the variability in study design [27]. The inputted data included sample size, outcome measures with their respective SD, and a correlation coefficient for within-subject measurements. These correlation coefficients were* r* = 0.65 and *r* = 0.49 for i-FABP and Lactulose/Rhamnose (L/R), respectively, based on previous data from our laboratory and the research team [28]. Due to the low number of studies using Iohexol (*n* = 1), plasma sugar probes (*n* = 3) or lipopolysaccharide (LPS) (*n* = 4), these were not meta-analysed, but are presented in the summary table. Meta-regression analyses and subgroup analysis were performed to investigate the effect of exercise duration, pre-exercise nutritional state (fasted or fed), timing of saccharide drink and environmental conditions on gut damage/permeability outcomes.

For data extraction, due to the similar size and structure of rhamnose (164.157 g/mol) and mannitol (182.172 g/mol), in the studies using urinary saccharide excretion assessment, these two urinary disaccharide/monosaccharides ratios were grouped together (DS/MS) as part of the meta-analysis. Intestinal fatty acid binding protein is a plasma marker of gut cell damage, and was the marker used most frequently across the studies. While some studies obtained multiple measures over time, the peak levels were observed directly after the completion of exercise, and these values were used for the meta-analysis. Gastrointestinal symptom data are reported as frequency and/or severity, using a variety of Likert scales (0–4, 1–5, 0–10) and visual analogue scales (mm). Studies were deemed to be fasted if the exercise trial was completed after an overnight fast.

Exercise data were extracted as duration (minutes) and intensity (various measures). Exercise intensity was not consistently presented as a percentage of maximal oxygen uptake (%*V*O_2max_) and in spite of various conversion equations available in the literature [29], it was not included as a moderator variable in the meta-regressions due to the concern that the transformation of multiple intensity measures was not mathematically robust. Exercise duration was used as a continuous variable in the meta-regression analysis. Where a study provided data for multiple arms that had different environmental conditions, exercise intensity or exercise duration, then the study trials were analysed separately to enable subgroup comparisons. However, multiple study arms with the same environmental conditions were pooled to prevent overpowering of a single study. The basic characteristics of the participants in the studies and the exercise protocols used were reported as median and an interquartile range [(IQR) lower and upper quartiles] due to the distribution of certain data in these studies.

The standardised mean difference (Hedges’ *g*) was interpreted as follows: < 0.20 as trivial, 0.2–0.39 as small, 0.40–0.80 as moderate and > 0.80 as large [30]. Therefore, a larger Hedges’ *g* would illustrate that exercise was associated with an increase in the selected outcome, while a value closer to zero would show a limited impact of exercise. Heterogeneity between the studies was assessed using the *I*-squared statistic, where 0–40% suggests heterogeneity might not be important, 30–60% may represent moderate heterogeneity, 50–90% may represent substantial heterogeneity and 75–100% represents substantial heterogeneity [31]. Sensitivity analysis was employed by omitting each study in turn. Subgroup analysis was performed for exercise performed in hot vs. thermoneutral environments (< 23 degrees Celsius [32]) A portion of the studies cited using standard laboratory conditions (no reported temperature), so it was assumed that this would be thermoneutral (19°–22°) as per laboratory standard practices. Other studies that targeted hot conditions reported conditions from 30° to 40°, thereby creating a naturally dichotomous outcome. Subgroup analysis was also performed for the fed or fasted state and the timing of the saccharide drink reported in different protocols as either before, during (at any point) or after (immediately) exercise.

### Exploration of Small Study Effects

Small study effects were explored with funnel plots of standard mean difference (SMD) vs. standard errors and by quantifying Egger’s linear regression intercept. A large and statistically significant Egger statistic indicates the presence of a small study effect [24].

## Results

**Fig. 1:**
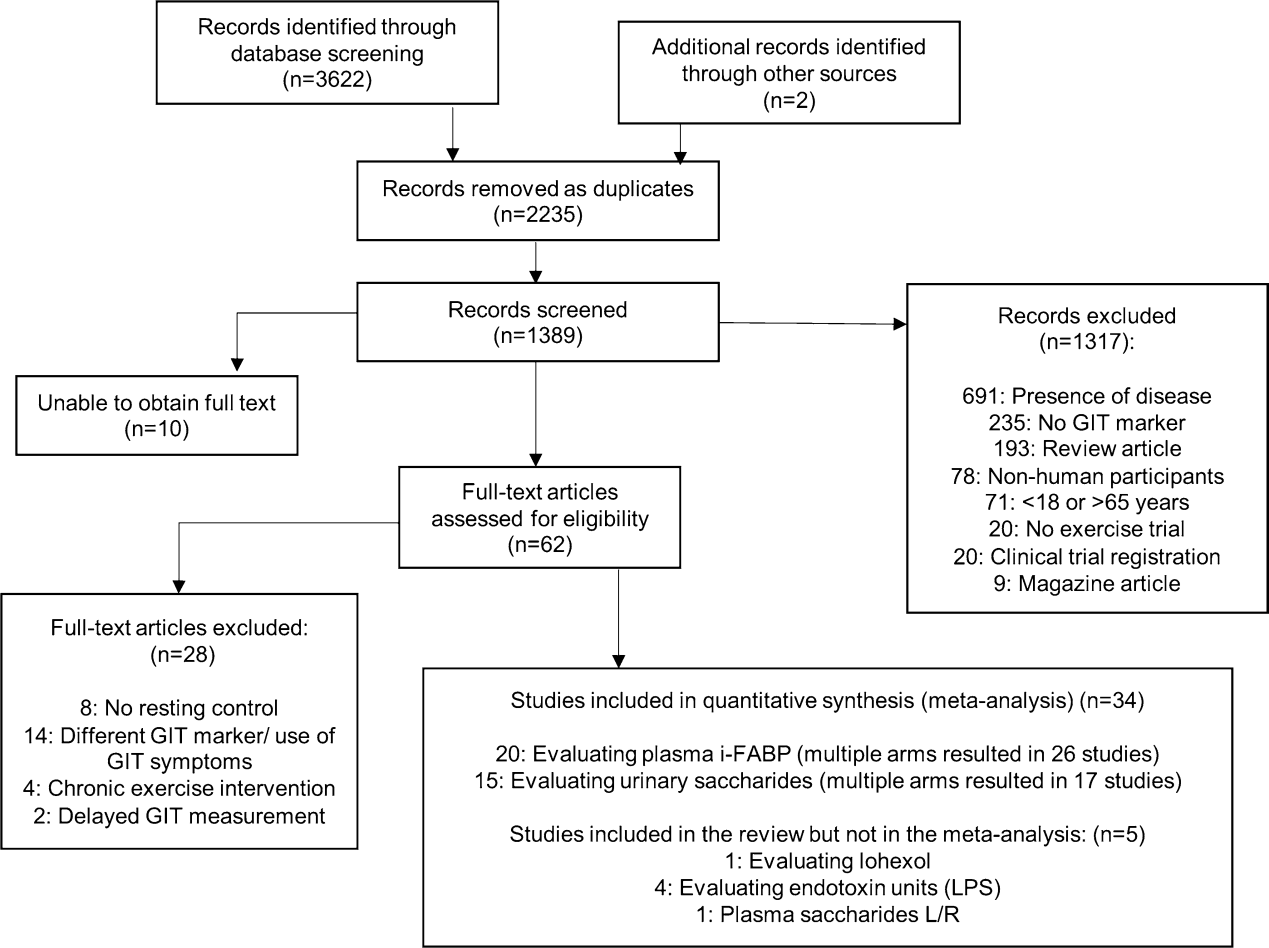
Flow chart of study selection [24]

In total, 34 studies met the inclusion criteria for the meta-analysis. Figure 1 outlines the flowchart of the study selection. All included studies had been published in peer reviewed scientific journals at the time of inclusion. Within the included studies, 20 studies measured plasma i-FABP [4, 9, 11, 12, 20, 33–47], while 15 studies measured the urinary L/R or L/M ratio (DS/MS) [7, 9, 11, 12, 18, 28, 36, 41, 48–53]. Studies assessing gut permeability via the urinary excretion ratio of lactulose to rhamnose/mannitol (L/R or L/M) provided a range of dosages from 1g (n = 2), 5g (n = 12) to 10g (n = 1) of lactulose in combination with 0.5–5g of rhamnose (or 2–5g of mannitol). Three studies assessed plasma L/R [9, 40, 54], and one used Iohexol [33]. Multiple arms of certain studies with different environmental conditions resulted in a higher number of conditions for i-FABP (n = 26) and DS/MS (n = 17) in the meta-analysis compared to total studies (raw data is presented in the supplementary material). Approximately half the studies were performed after an overnight fast (n = 17) [4, 11, 12, 18, 28, 36–38, 40, 46, 47, 49, 50, 52, 53, 55, 56], and reported GIS as part of the trials (n = 16) [7, 12, 18, 20, 33, 35, 42–45, 47, 48, 50, 51, 56, 57].

### Participant Characteristics Across the Studies

A total of 391 participants were included in this meta-analysis. Median (IQR) age across the studies was 26 years (IQR = 19–30) with 12% (48 of 391) of the participants being female. There were no sex-based differences in gut permeability cited in the included studies. Twenty-seven of the studies reported body mass: 74.6 kg (IQR 58.9–76.8), twenty-three studies reported height: 1.77 m (IQR 1.64–1.79). Other participant characteristics were not consistently reported across studies, but all subjects were classified as healthy. Only one study used untrained participants [39].

### Exercise Characteristics

Except for one study that used a resistance training protocol, all other studies included either running (*n* = 23) or cycling (*n* = 9) or a combination thereof (*n* = 1). The duration of exercise ranged from 20 to 328 min (median 60, IQR 60–90 min). Sixteen studies reported exercise intensity as a percentage of *V*O_2max_ (median 68, IQR 60–70% mL/min^−1^ kg^−1^), with seven studies reporting their steady state protocol as a percentage of maximal power (median 70, IQR 60–70% Watt_max_). Two studies took place outside over the course of a registered marathon [7, 33] (variable intensity), while three studies used intermittent protocols or other measures of intensity. The marathons were under race conditions outside, while the remaining were performed under laboratory conditions.

### Environmental Conditions Across the Studies

Half of the studies (10 of 20) measuring i-FABP [20, 37–40, 42–44, 50, 57] and 20% (3 of 15) of the studies measuring urinary DS/MS ratio [50, 52, 53] investigated responses to exercise in hot conditions (> 23 degrees centigrade) while the remainder were in thermoneutral or laboratory conditions (19–23 ˚C). One study (registered marathon) started at −2 ˚C, but no further temperature changes were stated [48].

### Other Measures

LPS was reported in four studies [33, 55, 57, 58]. Three of the four showed significant increases, but one study showed a significant increase in the hot condition only compared with the thermoneutral condition [58]. Iohexol was used in one study, and showed a simultaneous significant increase in permeability and gut damage in response to ninety minutes of running at an equivalent of 80% of 10 km personal best pace [33]. GIS of participants were reported in a variety of formats (Likert scales with 0–4, 1–5 and 0–10, visual analogue scales in millimetres). There was a large range of results. Some studies found no increase in symptoms around the exercise bout [35, 47], while others found a high frequency of symptoms reported [43, 59]. Supplementary table one provides details of the experimental protocols and raw data for each study.

### Meta-analysis

**Fig. 2:**
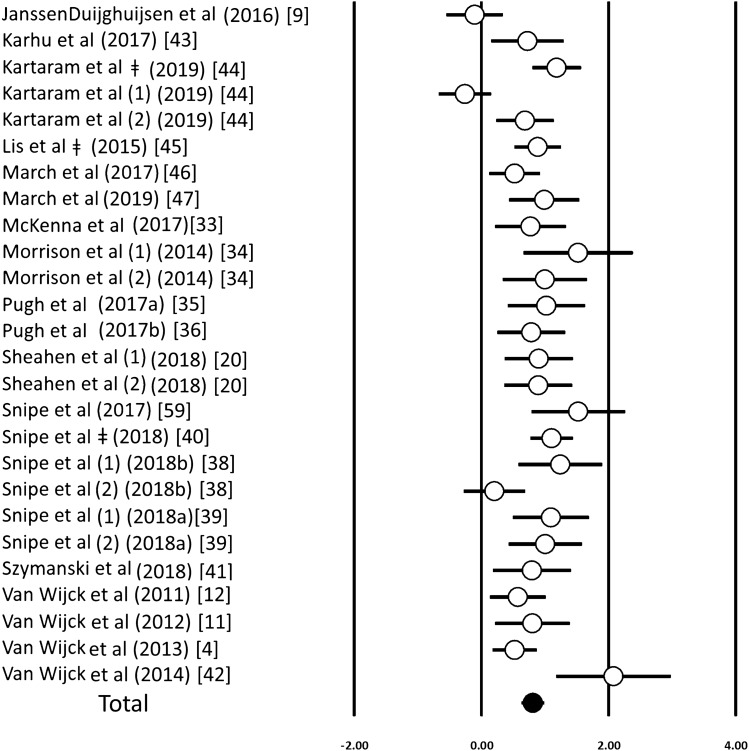
Forest plot of standardised mean differences (means ± 95% confidence intervals [CIs]) for studies evaluating the influence of an acute exercise bout on concentrations of plasma i-FABP. The solid circle represents the ES (mean ± 95% CI) for the model. Dagger: studies with multiple arms with similar environmental conditions that have been combined to prevent overpowering of the study; (1, 2) represent studies with multiple arms that were analysed separately

An acute bout of exercise induced a significant increase in circulating i-FABP concentrations (ES 0.81; 95% CI 0.63–0.98; *n* = 26; *p* < 0.001) (Fig. 2). The degree of heterogeneity may be substantial between these studies (*I*^2^ = 68.1%; *Q* = 78.4; *τ*^2^ = 0.13; *d*_f_ = 25). Inspection of the funnel plot and Egger’s regression intercept revealed evidence of small study effects (intercept 2.89; 95% CI 0.16–5.62; *p* = 0.03). Sensitivity analysis revealed minor changes only, and these changes did not substantially alter the overall mean effect. There was no effect of exercise duration using a meta-regression (*p* = 0.227). Using environmental temperature as a moderator in a subgroup analysis revealed a larger increase in i-FABP after exercise in a hot environment compared with lower temperatures [ES 1.06 (0.88–1.23) vs. 0.66 (0.43–0.89); *p* < 0.001]. This effect of heat remained significant after adjusting for exercise duration (*R*^2^ = 0.25, *p* = 0.02). When quantified via sub-group analysis, the fasted and fed state had no influence on these findings (Supplementary Table 4).

**Fig. 3:**
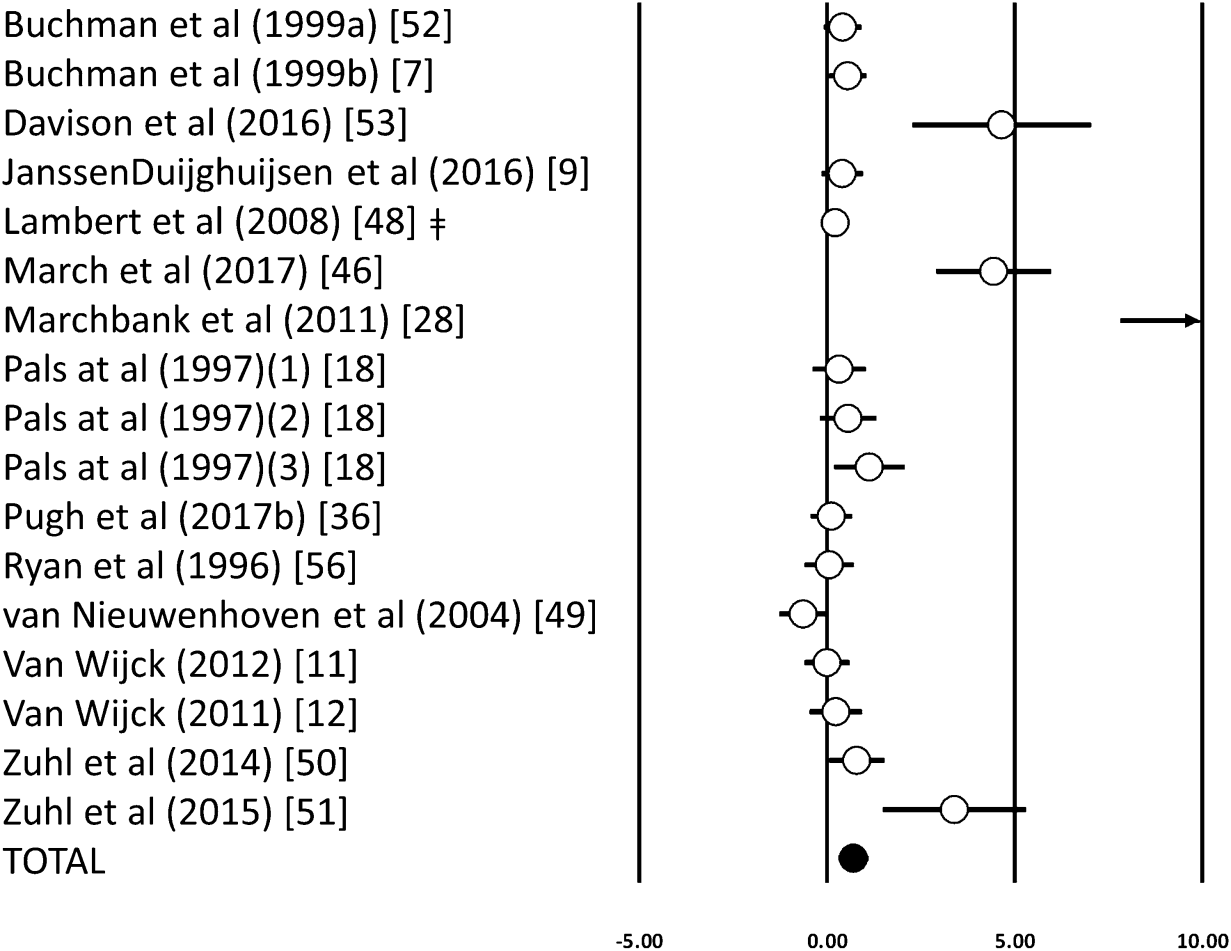
Forest plot of standardised mean differences (means ± 95% CIs) for studies evaluating the influence of an acute exercise bout on the ratio of Lactulose and Rhamnose or Lactulose and Mannitol in the urine (disaccharide/monosaccharide). The solid circle represents the ES (mean ± 95% CI) for the model. ǂ, Studies with multiple arms with similar environmental conditions that have been combined to prevent overpowering of the study; a,b, represents different studies from the same author published in the same year; (1, 2) represents studies with multiple arms that were analysed separately; → shows value outside of the figure area and can be found in the raw data in the supplementary material

Acute exercise induced a significant increase in the urinary DS/MS ratio (ES 0.70; 95% CI 0.29–1.11; *n* = 17; *p* < 0.001) (Fig. 3). The degree of heterogeneity may be substantial between these studies (*I*^2^ = 82%; *Q* = 90.1, *τ*^2^ = 0.61, *d*_f_ = 16). Inspection of the funnel plot and Egger’s regression intercept revealed evidence of small study effects (intercept 3.64, 95% CI 1.87–5.41; *p* < 0.001). Sensitivity analysis revealed minor changes only, and these changes did not substantially alter the overall mean effect. Using meta-regression analysis, there were no significant relationships between urinary DS/MS ratio and exercise duration (*p* = 0.29). There was a significant difference between the fasted or fed conditions [ES 1.16 (95% CI 0.53–1.79) vs. 0.20 (95% CI − 0.18 to 0.58), *p* = 0.01]. In addition, the timing of saccharide drink ingestion had a significant moderator effect on the urinary DS/MS ratio [before, ES 0.05 (95% CI − 0.34 to 0.45), during, ES 0.62 (95% CI 0.14 to 1.10) and after, ES 2.10 (95% CI 0.82–3.33) *p* = 0.005]. Only three studies measuring the DS/MS ratio were conducted in a hot environment, which prevented further subgroup analysis.

### Risk of Bias

**Fig. 4:**
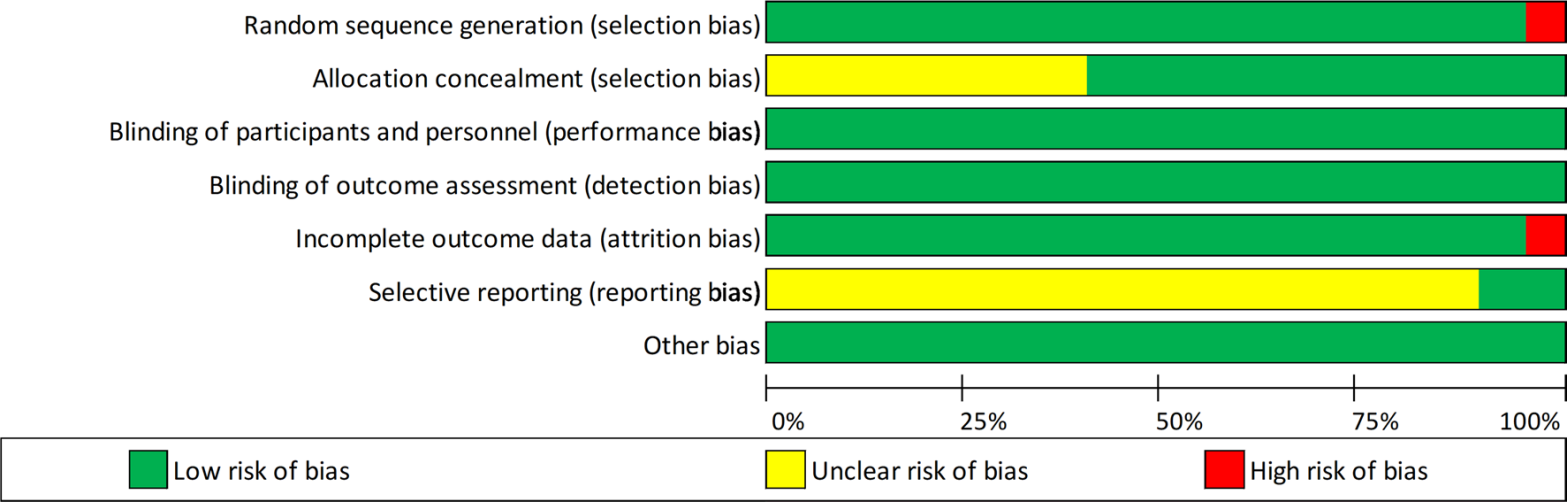
Summary of the risk of bias for all studies using the Cochrane tool [24]

Due to the randomised controlled design for the majority of the studies, the risk of bias was low or unclear. The risk of bias scores for the individual studies are presented in Supplementary Table 5, and an overall summary is displayed in Fig. 4.

## Discussion

The purpose of this meta-analysis was to examine the effect of an acute bout of exercise on markers of gut permeability and gut cell damage and the impact of the moderator variables exercise duration and environmental temperature. Overall, across the 34 included studies we observed a large and moderate effect size for increases in gut damage and gut permeability in response to a single bout of exercise, respectively. These findings support the current hypothesis that a bout of exercise is likely to induce damage to the endothelial lining and increase gut permeability in healthy people when compared with a resting control trial. This may have consequences for athletes and individuals engaging in strenuous exercise regimes by increasing the risk of endotoxaemia or impairing recovery via decreased nutrient absorption after exercise [4]. Appropriate nutritional strategies may be required to promote gut health in such scenarios.

Gut endothelial cell damage showed a large effect size in response to exercise. The studies that had effect sizes close to zero were those with lower intensity exercise protocols (50% *W*_max_) [9, 34]. Due to the different reporting of exercise intensity across the studies, this was not included in the meta-regressions to consider its impact; however, this would fall in line with different studies showing increased permeability at a higher intensity exercise [18]. I-FABP is found in the top layer of the endothelial cells and plasma-based increases confirm damage to the endothelial cells themselves, rather than the tight junctions. Such damage is attributed to the hypoxia-related changes in pH, oxidative stress, mechanical stress and accumulating metabolite levels [60]. Gut cell damage (as indicated by plasma [i-FABP]) may reflect a deterioration of all transcellular transport proteins and imply a consequent impairment to nutrient transport capacity and nutrient absorption. To date, this has been assessed in a single study investigating protein absorption in relation to the change in gut damage markers post-exercise [4]. The study found a significant correlation between intestinal injury (i-FABP) and dietary protein digestion and absorption rates post-exercise (*r* = − 0.62, *p* < 0.05). Another study using 90 min of cycling at 50% maximal power showed a lower plasma amino acid level post exercise compared to rest [9] but no associations were made with gut damage markers. Interestingly, i-FABP levels across studies tend to return to baseline levels within 2–4 h of exercise [41, 42]. However, this may indicate clearance of the endothelial protein from the systemic circulation rather than full repair or maturation of premature enterocytes to restore the endothelial lining [61]. The half-life of i-FABP is estimated at eleven minutes, which is significantly shorter than healthy enterocyte turnover. Ischemia/reperfusion has also been linked to the apoptosis of Paneth cells in the crypt of the villi which will not be illustrated by i-FABP levels [62]. The timeline of cell damage and its associated impact may warrant further investigation considering the large focus in sports nutrition on the rapid introduction of nutrition and hydration during the recovery period [63].

Gut permeability showed a moderate effect size in response to exercise. This is in line with the current evidence showing that exercise-associated gut hypoxia, reperfusion and resulting oxidative damage may disrupt the upper gastrointestinal tight junction proteins between endothelial cells and allow for increased paracellular transport [49]. The effects of exercise are reasonably consistent across the studies; however, there were four studies that had a considerably larger magnitude of change compared to the remaining studies. Of the four studies, three use the same research protocol, exercise test, analysis methods and equipment (communication from the author). Due to the lack of significant findings in the meta-regression, it is difficult to determine if the exercise protocol itself (20 min of running at 80% *V*O_2max_) or methods of analysis and timing of the saccharide drink have contributed to the differences found. Studies that used the MS/DS test to measure permeability had a variation of dosages (e.g., 1, 5, or 10 g lactulose) and larger dosages have been associated with changes in post-absorption kinetics which can reflect in a lower urinary ratio of the sugars [14]. The studies that tested participants after an overnight fast showed larger changes in the disaccharide ratio which is in line with findings from Snipe et al., where feeding carbohydrate or protein prior to exercise diminished the gut damage and permeability compared to water [59]. This is most likely due to the lower levels of splanchnic hypoxia in the fed state as well as the availability of adenosine triphosphate. This is in contrast to the lack of influence of the fasted state on the change in i-FABP. The timing of the saccharide drink showed that studies, where the drink was given immediately after exercise showed the highest levels of change in the DS/MS ratio. A drink administered prior to exercise may pass through the upper gastrointestinal tract prior to damage occurring. Exercise itself may also result in reduced urine output which plays a role in the recovery percentage of the sugars [3, 64] Markers of gut damage or permeability are often validated using anti-inflammatory medications [65], or in diseased populations [66] which may hinder some of the interpretation in exercise studies, and show the importance of a standardised protocol being introduced in athlete populations in future research [67]

Increased permeability and gut cell damage may allow for increased bacterial translocation, causing an acute localised inflammatory reaction [68]. The four identified studies that measured plasma LPS showed an increase post-exercise compared to pre-exercise levels [33, 55, 57, 58] (data presented in Supplementary Table 1). While this sample of studies is too small to analyse or draw conclusions, these data support the view that endurance style exercise may increase the risk of endotoxaemia for individuals via increased gut permeability [22]. Using an alternative methodology, March et al. added supporting data showing a significant association between post-exercise gut damage (i-FABP) and the ratio of plasma bacteroides/total bacterial DNA, to illustrate increased bacterial translocation [36]. Selected studies have also shown an increase in inflammatory markers post-exercise, although this was beyond the scope of the present meta-analysis [43, 52, 60]. Such additional data continues to support the exercise permeability cascade resulting in increased risk of bacterial translocation post-exercise [3, 6]. This may be particularly relevant for athletes during high training volumes or during events, where gastrointestinal illness and general illness risk is known to be elevated [69].

Gut permeability and gut damage outcomes had no significant interactions with exercise duration. All studies had exercise protocols lasting twenty minutes or longer, which makes them likely to induce similar splanchnic hypoxia [12]. It is likely that even in the longer marathon events that due to the variable intensity there was no measurable difference between the studies once hypoxia has occurred. Conversely, gut damage had a significant relationship with hot conditions (> 23 °C). A systematic review by Pires et al. found a significant correlation between L/R ratio (gut permeability) and core body temperature, showing an exponential increase in permeability once core temperature was elevated > 39 °C [21]. Hot conditions cause further diversion of blood supply to the cutaneous vascular bed to increase body heat loss, perpetuating the splanchnic hypoperfusion [52]. Fluid loss as part of thermoregulation contributes to dehydration, which in turn may also contribute to increased hypoperfusion and further permeability [50]. Although the present meta-analysis was not able to investigate the effect of environmental temperature on gut permeability due to an insufficient number of studies being available, the significantly greater increase in gut damage during exercise in hot conditions adds to this previous literature and suggests that strategies to minimise damage may be particularly important when exercising in hot environments.

It is worth noting that heat, dehydration and moderate to high intensity exercise may all exist simultaneously for athletes during events. Due to the majority of the studies being focussed on steady state endurance exercise in laboratory settings, there is limited understanding of gut outcomes in other scenarios. Pugh et al. assessed 400 m repeated running intervals at 120% of *V*O_2max_, but more research is required around other intermittent team sports like hockey, soccer or intermittent combat/contact sports like rugby or American football. There are also few studies that have measured changes in gut permeability in response to a chronic exercise stimulus, meaning that further research around the cumulative nature of high intensity training on gut health, illness risk, nutrient absorption, or repeated bouts of exercise (such as during a tournament) is required.

The risk of bias within the studies included in this meta-analysis was low or unclear due to the majority of the studies using a randomised crossover design. However, the large variation in the methodologies used created limitations for the meta-analysis. This includes a range of exercise protocols being implemented with different styles of reporting within manuscripts which made the investigation of exercise intensity unsuitable for meta-regression analysis. While a number of studies presented their power calculations around sample size, the small sample sizes in the included studies is an ongoing limitation. Additionally, while the gut permeability cascade is well supported by evidence, robust data linking the biochemical changes to GIS in athletes is lacking. Studies that have performed statistical analysis of GIS and biochemical markers found no [8], or weak relationships, or compare placebo to nutraceutical arms [42, 57]. A study in combat soldiers found a significant correlation between GIS and urinary metabolite profiles, which in turn were correlated to permeability measures [70]. This likely demonstrates the complex aetiology of gut symptoms, and that any such relationships are unlikely to be best described by simple linear correlations, with multiple (nutritional, pharmacological, psychological and physiological) contributing factors.

## Conclusion

This meta-analysis confirms that a single bout of exercise causes significant increases in gut damage and gut permeability in healthy participants. Furthermore, the gut damage induced by exercise is exacerbated in hot environments. Long-term and pre-exercise nutritional strategies may play a role in reducing the damage incurred by exercise [40, 42, 57], but further research is required into the efficacy of nutritional strategies during the post-exercise period once damage and increased permeability have already occurred.

## Electronic supplementary material

Below is the link to the electronic supplementary material.

Supplementary file1 (DOCX 167 kb)

Supplementary file2 (DOCX 13 kb)

## Declarations

### Data availability

Data sharing not applicable to this article. All data extracted and raw meta-analysis are presented in tables one through five in the supplementary material.
