# Supplementary material for: The Effects of Exercise on Indirect Markers of Gut Damage and Permeability: A Systematic Review and Meta-analysis
Source: Sports Med. 2020 Nov 17;51(1):113–24. doi: 10.1007/s40279-020-01348-y (PMC7806566; doi:10.1007/s40279-020-01348-y)
Supplement: Supplementary file 1 — Supplementary file1 (DOCX 167 kb) [file 40279_2020_1348_MOESM1_ESM.docx]

Article title: The effects of exercise on indirect markers of gut damage and permeability: a systematic review and meta-analysis

Journal: Sports Medicine

Authors names: Sarah Chantler, Alex Griffiths, Jamie Matu, Glen Davison, Ben Jones, Kevin Deighton

Corresponding author: Sarah Chantler, Carnegie Applied Rugby Research Centre, Institute for Sport, Physical Activity and Leisure, Leeds Beckett University, Leeds, LS6 3QS, United Kingdom (email: [s.a.chantler@leedsbeckett.ac.uk](mailto:s.a.chantler@leedsbeckett.ac.uk))

**Supplementary table 1: Basic information for studies included in the meta-analysis and systematic review**

| **Reference** | **n** | **Age (years)** | **Body Mass (kg)** | **Exercise intervention** | | | | **Temp (˚C)** | **Measured outcomes**  **Mean pre exercise (SD) – Mean post exercise (SD)** | | |
| --- | --- | --- | --- | --- | --- | --- | --- | --- | --- | --- | --- |
|  |  |  |  | Mode | Duration (minutes) | Reported intensity (various measures) | Fed/ Fasted |  | Urinary DS/MS (% excretion) | Plasma  i-FABP (pg/mL) | Other measures |
| Ashton et al 2003 [55] | 10 | 18 – 30 | 79.9 (4.2) | Cycle ergometer | 15.17 (1.01) | Incremental TTE | Fasted | Not reported | - | - | LPS (Eu/ml) 0.14 (0.09) - 0.24 (0.19)^#^ |
| Buchman et al 1999a [52] | 10 | 18-55 | Not reported | Road marathon (Houston- Methodist Marathon) | 215 ± 28.3 | Variable | n/a | -2˚C (start of race) | 0.02 (0.01) – 0.03 (0.02)^c^ (n=6) | - | GIS (scale 1-5) no occurrence during the race. Heme-positive stool 2/10 runners. |
| Buchman et al 1999b [7] | 15 | 25-49 | Not reported | Road marathon (Houston-Tenneco Marathon) | 173-328 | Variable | n/a | Not reported | 0.03 (0.02) – 0.07 (0.10)^c^ | - | GIS (scale 1-5), Cramping (53%), pain (33%), nausea (47%) reported. Heme-positive stool in 2/10 runners |
| Davison et al 2016 [53] | 8 | 25 | 80.1 (7.1) | Treadmill running at 1% gradient | 20 | 80% VO_2max_ | Fasted | Not reported | 0.02 (0.01) – 0.06 (0.01)^c #^ | - | - |
| Janssen Duijghuijsen et al 2016 [9] | 11 | 27 (4) | 77.8 (9.4) | Cycle ergometer | 90 | 50% W_max_ | Fed | Not reported | 0.02 (0.01) – 0.02 (0.01)^a^ | 224 (63) – 214 (96) | Serum L/R (%)  0.05 (0.02) – 0.11 (0.06) |
| Janssen Duijghuijsen et al 2017 [54] | 11 | 27 (4) | Not reported | Cycle ergometer | 90 | 50% W_max_ | Fed | 23 ˚C | - | - | § Serum L/R (%)  0.05 (0.03) – 0.11 (0.06)^#^ |
| Karhu et al 2017 [43] | 9 | 35(6) | Not reported | Treadmill running | 90 | 80% of 10km personal best (5:12 ± 0.59 min/km) | n/a | Not reported | - | 314 (152) – 804 (599)^#^ | Iohexol (%)  0.20 (0.18) – 0.39 (0.24)^#^  LPS (Eu/ml) 0.57 (0.12) – 0.66 (0.17)  GIS: (frequency during and after exercise) 5/9 reported at least one symptom ; 4/9 flatulence, 1/9 burping, 1/9 diarrhoea; 1/9 constipation |
| Kartaram et al 2019 (1) ǂ [44] | 15 | 24 (2) | 75.8 (6.7) | Cycle ergometer (with water) | 60 | 70% W_max_ | Fed | Not reported | - | 742 (341) – 1262 (512) | - |
|  |  |  |  | Cycle ergometer (no water) | 60 | 70% W_max_ | Fed | Not reported | - | 689 (310) – 1559 (658) ^#^ | - |
| Kartaram et al 2019 (2) [44] | 15 | 24 (2) | 75.8 (6.7) | Cycle ergometer | 60 | 55-85% W_max_ in 2 min intervals (mean 70% W_max_) | Fed | Not reported | - | 698 (369) – 598 (360) | - |
| Kartaram et al 2019 (3) [44] | 15 | 24 (2) | 75.8 (6.7) | Cycle ergometer | 60 | 50% W_max_ | Fed | Not reported | - | 720 (359) – 1198 (714) | - |
| Lambert et al 2008 ǂ [48] | 20 | 22 (3) | Not reported | Treadmill running with water | 60 | 70 %VO_2max_ | Fasted | 24˚C | 0.05 (0.07) – 0.06 (0.08)^a^ | - | GIS: (VAS mm) no significant increase in symptoms during exercise |
|  |  |  |  | Treadmill running without water | 60 | 70 %VO_2max_ | Fasted | 24˚C | 0.05 (0.07) – 0.08 (0.12)^a^ ^#^ | - | GIS: (VAS mm) no significant increase in symptoms during exercise |
| Lis et al 2015 ǂ [45] | 13 | 32 (7) | 71.1 (13.4) | Cycle ergometer (gluten) | 60 | 45 min steady state (70% W_max_) with 15 min TT | Fed | 20 ˚C | - | 94 (83) – 304 (191) | GIS (scale 0-10): no significant increase in symptoms during exercise |
|  |  |  |  | Cycle ergometer (gluten free) | 60 | 45 min steady state (70% W_max_) with 15 min TT | Fed | 20 ˚C | - | 99 (57) – 301 (252) | GIS (scale 0-10): no significant increase in symptoms during exercise |
| March et al 2017 [46] | 18 | 26 (5) | 77.0 (9.8) | Treadmill running 1% gradient | 20 | 80% VO_2max_ | Fasted | 22˚C | 0.35 (0.06) – 0.95 (0.15)^c #^ | 578 (653 ) – 928 (625)^#^ | - |
| March et al 2019 [47] | 12 | 26 (6) | 79.5 (9.4) | Treadmill running | 60 | 70% VO_2max_ | Fasted | 30˚C | - | 749(349) – 2089 (1252)^#^ | - |
| Marchbank et al 2011 [28] | 12 | 26 | 74.7 (11.6) | Treadmill running at 1% gradient | 20 | 80% VO_2max_ | Fasted | Not reported | 0.02 (0.00) – 0.04 (0.00)^c #^ | - | - |
| McKenna et al 2017 [33] | 10 | 20 (2) | 76.8 (9.7) | Treadmill running | 46 ± 7.7 | 64% VO_2max (_95% ventilatory threshold) | Fasted | 40˚C | - | 851 (451 ) – 1267 (522)^#^ | - |
| Morrison et al 2014(1) [34] | 7 | 24 (4) | 74.6 (5.2) | Cycle ergometer and treadmill running (Trained) | 90 | 15 min cycle (41%VO_2peak_), 30 mins run (75% VO_2peak_), 30 mins TT run (75% VO_2peak_), 15 mins cycle (52% VO_2peak_) | Fed | 30 ˚ C | N/A^a^ | 143 (59) – 949 (423)^#^ | - |
| Morrison et al 2014(2) [34] | 8 | 21 (2) | 82.8 (16.5) | Cycle ergometer and treadmill running (Untrained) | 90 | 15 min cycle (41%VO_2peak_), 30 mins run (75% VO_2peak_), 30 mins TT run (75% VO_2peak_), 15 mins cycle (52% VO_2peak_) | Fed | 30 ˚ C | N/A^a^ | 160 (93) – 443 (260)^#^ | - |
| Pals et al 1997 (1) [18] | 6 | 30 (2) | Not reported | Treadmill running | 60 | 40% VO_2max_ | Fasted | 22˚ C | 0.05 (0.02) – 0.06 (0.01)^b^ | - | GIS (VAS mm) No significant GIS reported during exercise |
| Pals et al 1997 (2) [18] | 6 | 30 (2) | Not reported | Treadmill running | 60 | 60% VO_2max_ | Fasted | 22˚ C | 0.05 (0.02) – 0.06 (0.02)^b^ | - | GIS (VAS mm) No significant GIS reported during exercise |
| Pals et al 1997 (3) [18] | 6 | 30 (2) | Not reported | Treadmill running | 60 | 80% VO_2max_ | Fasted | 22˚ C | 0.05 (0.02) – 0.11 (0.05)^b #^ | - | GIS (VAS mm):1/6 experienced abdominal cramping and side ache during exercise |
| Pugh et al 2017a [35] | 10 | 24 (4) | 74.7 (8.5) | Treadmill running | 60 | 70% VO_2max_ | Fasted | 30˚C |  | 352 (204) – 602 (238) | Serum L/R (%) 0.02 (0.01) – 0.09 (0.03)^#^  Urinary Sucrose (%)  0.12 (0.12) - 0.17 (0.17)  Serum Sucrose (%)  0.14 (0.14) – 0.39 (0.22) |
| Pugh et al 2017b [36] | 11 | 33 (10) | 75.1 (5.8) | Interval running | ~45 | 3 x 6 (400m) at 120% VO_2max_ and 50%_max_ recovery for 75% of 400m time with 3 mins rest in between | Fed | 23˚C | 0.03 (0.02) – 0.03 (0.02)^c ᵦ^ | 481 (334)–829 (448)^#^ |  |
| Ryan et al 1996 [56] | 7 | 29 (8) | 71.6 (5.4) | Treadmill running | 60 | 65% VO_2max_ | Fasted | 22 ˚C | 0.01 (0.01) – 0.03 (0.03)^a^ | - | GIS (VAS mm): urge to defecate was 14.1±6% in exercise vs 0.4±0.2% at rest (NS). Severity of symptoms as <10% |
| Shing et al 2014 [57] | 10 | - | 71.5 (7.2) | Treadmill running | 33 ± 2.2 | 80% of ventilatory threshold TTE | Fed | 35˚C | - | - | LPS (Eu/ml)  0.16 (0.08) – 0.21 (0.08)^#^  GIS: 1.6±0.3 during exercise (no data at rest) |
| Sheahen et al 2018 (1) [20] | 12 | Not reported | 81.9 (7.9) | Cycle ergometer | 45 | 70% VO_2max_ | n/a | 20˚C | - | 571 (175) – 852 (317) | GIS (scale 0-4): No difference in severity during exercise |
| Sheahen et al 2018 (2) [20] | 12 | Not reported | 81.9 (7.9) | Cycle ergometer | 45 | 70% VO_2max_ | n/a | 30˚C | - | 585 (188) – 954 (411) | GIS (scale 0-4): No difference in severity during exercise |
| Snipe et al 2017 [59] | 11 | 31 (5) | 65.7 (12) | Treadmill running | 120 | 60% VO_2max_ | Fed | 35˚C | N/A^b^ | 894 (451) from baseline^#^ | GIS (scale 0-10): 82% reported symptoms with severity >5/10 |
| Snipe et al 2018a (1) [39] | 10 | 31 (6) | 65.7 (12) | Treadmill running | 120 | 60% VO_2max_ | Fed | 22˚C | - | 276 (196) from baseline | GIS(scale 0-10): 70% of participants reported symptoms |
| Snipe et al 2018a (2) [39] | 10 | 31 (6) | 65.7 (12) | Treadmill running | 120 | 60% VO_2max_ | Fed | 30˚C | - | 581 (449) from baseline^#^ | GIS(scale 0-10): 80% of participants reported symptoms |
| Snipe et al 2018b (1) [38] | 10 | 31 (6) | 66.3 (10.5) | Treadmill running | 120 | 60% VO_2max_ | Fed | 22˚C | - | 274 (212) from baseline | GIS(scale 0-10): 70% of participants reported symptoms |
| Snipe et al 2018b (2) [38] | 10 | 31 (6) | 66.3 (10.5) | Treadmill running | 120 | 60% VO_2max_ | Fed | 35˚C | - | 1230 (727) from baseline^#^ | GIS(scale 0-10): 90% of participants reported symptoms |
| Snipe et al 2018c ǂ[40] | 12 | 37 (8) | 66.7 (10.7) | Treadmill running with cold water | 120 | 60% VO_2max_ | Fed | 35˚C | - | 1240 (918) from baseline^#^ | GIS(scale 0-10): 75% of participants reported symptoms |
|  |  |  |  | Treadmill running with cool water | 120 | 60% VO_2max_ | Fed | 35˚C | - | 1270 (987) from baseline^#^ | GIS(scale 0-10): 92% of participants reported symptoms |
|  |  |  |  | Treadmill running with temp water | 120 | 60% VO_2max_ | Fed | 35˚C | - | 1710 (1046) from baseline^#^ | GIS(scale 0-10): 92% of participants reported symptoms |
| Szymanski et al 2018 [41] | 8 | 19 | 75.0 (15.8) | Treadmill running | 60 | 68% VO_2max_ | Fasted | 36.8˚C | - | 874 (350) – 1620 (876)^#^ |  |
| Van Nieuwenhoven et al 2004 [49] | 10 | 18-21 | 25-50 | Cycle ergometer (asymptomatic, hydrated) | 90 | 70% W_max_ | Fed | 19˚C | 0.02 (0.01) – 0.01 (0.01)^a #^ |  | GIS (scale 0-10): Exercise did not increase symptoms, but there was a correlation between Δnausea and Δgastric emptying (p=0.04) |
| Van Wijck et al 2011 [12] | 15 | 25 | Not reported | Cycle ergometer | 60 | 70% W_max_ | Fasted | Not reported | 0.03 (0.01) – 0.04 (0.03)^b^ (n=6) | 309 (206) – 615 (528)^#^ (n=15) | GIS (frequency during exercise): 3 participants reported minor symptoms during exercise |
| Van Wijck, et al 2012 [11] | 9 | 27 (1) | Not reported | Cycle ergometer | 60 | 70% W_max_ | Fasted | Not reported | 0.02 (0.04) – 0.02 (0.02)^b ᵦ^ | 295 (138) – 474 (222)^#^ | - |
| Van Wijck et al 2013 [4] | 24 | 21 | 77.0 (2.1) | Resistance training | 30 | Variable | Fasted | Not reported | - | 254 (107) – 344 (184)^#’^ | - |
| Van Wijck et al 2014 [42] | 10 | 25 (1) | Not reported | Cycle ergometer | 60 | 70% W_max_ | Fasted | Not reported | - | 172%(23) from baseline^#^ | GIS (frequency during and after exercise) : No symptoms reported |
| Yeh et al 2013 [58] | 15 | Not reported | 65.7 (7) | Treadmill running | 60 | 70% VO_2max_ | Fed | 25˚C | - | - | LPS 12.0 (6.4) – 10.9 (5.4) |
|  |  |  | 65.7 (7) | Treadmill running | 60 | 70% VO_2max_ | Fed | 33˚C | - | - | LPS 11.6 (4.3) – 16.6 (3.9)^#^ |
| Zuhl, et al 2013 [50] | 8 | 25 (4) | 72.2 (16.4) | Treadmill running | 60 | 71.3% VO_2max_ | Fasted | 30˚C | 0.02 (0.01) – 0.06 (0.04)^b #^ | - | - |
| Zuhl et al 2014 [51] | 7 | 26 (4) | 60.3 (6.7) | Treadmill running | 60 | 69.7% VO_2max_ | Fasted | 30˚C | 0.02 (0.01) – 0.06 (0.01)^b #^ | - | - |

DS/MS, disaccharide to monosaccharide ratio; a, b, c, multiple studies by the same author published in the same year; W_max,_ maximal power output in Watts; LPS, lipopolysaccharide; HRR, heart rate range; TTE, time to exhaustion; (1),(2) studies that included multiple arms that were analysed separately due to different environmental or exercise conditions; ǂ studies that contain multiple arms with similar environmental or exercise conditions (e.g. with or without water at the same temperature and intensity) that were pooled to prevent statistical overpowering; ^#^ studies that reported a significant change pre/rest to post exercise, p<0.05, ^a,b,c^, studies where the sugar probe solution was given prior^a^, during^b^, or after^c^ exercise; GIS, gastrointestinal symptoms; Fasted denotes any experimental procedures that commenced after an overnight fast; ^ᵦ^, denotes studies that collected urine at the 2 hour point compared to others that were 5 hours.

**Supplementary table 2: Raw statistical data for gut damage (i-FABP) from studies included in the meta-analysis**

| Statistics for each study | | | | | | | | | | | |
| --- | --- | --- | --- | --- | --- | --- | --- | --- | --- | --- | --- |
| Study name | Hedges's g | Standard error | Variance | Lower limit | Upper limit | Z-Value | p-Value | Relative weight | Std Residual | Sample Size |  |
| JanssenDuijghuijsen et al 2016 [9] | -0.11 | 0.23 | 0.05 | -0.55 | 0.34 | -0.47 | 0.64 | 4.32 | -2.16 | 12 |  |
| Karhu et al 2018 [43] | 0.72 | 0.29 | 0.08 | 0.15 | 1.29 | 2.49 | 0.01 | 3.67 | -0.19 | 9 |  |
| Kartaram et al 2018 (1) [44] | -0.26 | 0.21 | 0.04 | -0.67 | 0.15 | -1.24 | 0.21 | 4.51 | -2.57 | 15 |  |
| Kartaram et al 2018 (2) [44] | 0.69 | 0.23 | 0.05 | 0.23 | 1.14 | 2.97 | 0.00 | 4.28 | -0.29 | 15 |  |
| Kartaram et al 2018 (3) ǂ [44] | 1.18 | 0.19 | 0.04 | 0.80 | 1.56 | 6.09 | 0.00 | 4.66 | 0.92 | 30 |  |
| Lis et al 2015 ǂ [45] | 0.88 | 0.19 | 0.03 | 0.52 | 1.25 | 4.74 | 0.00 | 4.74 | 0.19 | 26 |  |
| March et al 2017 [46] | 0.52 | 0.20 | 0.04 | 0.13 | 0.92 | 2.58 | 0.01 | 4.57 | -0.69 | 18 |  |
| March et al 2019 [47] | 0.99 | 0.28 | 0.08 | 0.43 | 1.54 | 3.50 | 0.00 | 3.76 | 0.39 | 12 |  |
| McKenna et al 2017 [33] | 0.77 | 0.28 | 0.08 | 0.22 | 1.33 | 2.73 | 0.01 | 3.75 | -0.08 | 10 |  |
| Morrison et al 2014 (1) [34] | 1.52 | 0.44 | 0.19 | 0.66 | 2.38 | 3.46 | 0.00 | 2.47 | 1.25 | 7 |  |
| Morrison et al 2014 (2)[34] | 1.00 | 0.34 | 0.11 | 0.34 | 1.66 | 2.96 | 0.00 | 3.25 | 0.38 | 8 |  |
| Pugh et al 2017a [35] | 1.02 | 0.31 | 0.10 | 0.41 | 1.63 | 3.30 | 0.00 | 3.50 | 0.45 | 10 |  |
| Pugh et al 2017b [36] | 0.78 | 0.27 | 0.07 | 0.25 | 1.32 | 2.87 | 0.00 | 3.85 | -0.06 | 11 |  |
| Sheahen et al 2018 (1) [20] | 0.90 | 0.27 | 0.07 | 0.37 | 1.44 | 3.30 | 0.00 | 3.84 | 0.21 | 12 |  |
| Sheahen et al 2018 (2) [20] | 0.89 | 0.27 | 0.07 | 0.36 | 1.43 | 3.27 | 0.00 | 3.85 | 0.19 | 12 |  |
| Snipe et al 2017 [59] | 1.52 | 0.38 | 0.14 | 0.79 | 2.26 | 4.06 | 0.00 | 2.93 | 1.37 | 11 |  |
| Snipe et al 2018b (1) [38] | 1.24 | 0.34 | 0.11 | 0.58 | 1.90 | 3.69 | 0.00 | 3.25 | 0.88 | 10 |  |
| Snipe et al 2018b (2) [38] | 0.20 | 0.25 | 0.06 | -0.28 | 0.69 | 0.83 | 0.41 | 4.12 | -1.39 | 10 |  |
| Snipe et al 2018c ǂ[40] | 1.10 | 0.17 | 0.03 | 0.77 | 1.44 | 6.47 | 0.00 | 4.90 | 0.74 | 36 |  |
| Snipe et al 2018a (1) [39] | 1.09 | 0.30 | 0.09 | 0.49 | 1.69 | 3.58 | 0.00 | 3.54 | 0.60 | 10 |  |
| Snipe et al 2018a (2) [39] | 1.00 | 0.29 | 0.09 | 0.43 | 1.58 | 3.40 | 0.00 | 3.63 | 0.42 | 10 |  |
| Szymanski et al 2018 [41] | 0.79 | 0.31 | 0.10 | 0.18 | 1.40 | 2.54 | 0.01 | 3.47 | -0.03 | 8 |  |
| Van Wijck et al 2011 [12] | 0.57 | 0.22 | 0.05 | 0.13 | 1.01 | 2.56 | 0.01 | 4.36 | -0.56 | 15 |  |
| Van Wijck et al 2012 [11] | 0.80 | 0.30 | 0.09 | 0.22 | 1.39 | 2.69 | 0.01 | 3.60 | -0.02 | 9 |  |
| Van Wijck et al 2013 [4] | 0.52 | 0.18 | 0.03 | 0.17 | 0.87 | 2.94 | 0.00 | 4.83 | -0.72 | 24 |  |
| Van Wijck et al 2014 [42] | 2.08 | 0.46 | 0.21 | 1.18 | 2.98 | 4.52 | 0.00 | 2.34 | 2.17 | 10 |  |
| **Total** | **0.81** | **0.09** | **0.01** | **0.63** | **0.99** | **8.96** | **<0.001** |  |  |  |  |

a, b, or c, multiple studies by the same author published in the same year; (1),(2) studies that included multiple arms that were analysed separately due to different environmental or exercise conditions; ǂ studies that have multiple arms with similar environmental or exercise conditions that are pooled for the meta-analysis to prevent overpowering

**Supplementary table 3: Raw data for gut permeability (Disaccharide/monosaccharide ratio) from studies included in the meta-analysis**

| Statistics for each study | | | | | | | | | | | |
| --- | --- | --- | --- | --- | --- | --- | --- | --- | --- | --- | --- |
| Study name | Hedges's g | Standard error | Variance | Lower limit | Upper limit | Z-Value | p-Value | Relative weight | Std Residual | Sample size |  |
| Buchman et al 1999b [7] | 0.42 | 0.26 | 0.07 | -0.09 | 0.92 | 1.61 | 0.11 | 7.43 | -0.39 | 15 |  |
| Buchman et al 1999a [52] | 0.55 | 0.25 | 0.06 | 0.06 | 1.05 | 2.19 | 0.03 | 7.47 | -0.21 | 10 |  |
| Davison et al 2016 [53] | 4.66 | 1.22 | 1.48 | 2.27 | 7.04 | 3.82 | 0.00 | 2.15 | 2.83 | 8 |  |
| JanssenDuijghuijsen et al 2016 [9] | 0.41 | 0.28 | 0.08 | -0.15 | 0.96 | 1.43 | 0.15 | 7.26 | -0.40 | 12 |  |
| Lambert et al 2008ǂ [48] | 0.22 | 0.16 | 0.02 | -0.09 | 0.52 | 1.41 | 0.16 | 8.02 | -0.69 | 40 |  |
| March et al 2017 [46] | 4.44 | 0.78 | 0.61 | 2.91 | 5.97 | 5.68 | 0.00 | 3.82 | 3.60 | 18 |  |
| Marchbank et al 2011 [28] | 17.77 | 3.67 | 13.49 | 10.57 | 24.97 | 4.84 | 0.00 | 0.31 | 4.57 | 12 |  |
| Pals at al 1997 (1) [18] | 0.33 | 0.36 | 0.13 | -0.38 | 1.03 | 0.90 | 0.37 | 6.70 | -0.49 | 6 |  |
| Pals at al 1997 (2) [18] | 0.56 | 0.38 | 0.15 | -0.19 | 1.31 | 1.46 | 0.14 | 6.52 | -0.18 | 6 |  |
| Pals at al 1997 (3) [18] | 1.13 | 0.48 | 0.23 | 0.19 | 2.07 | 2.36 | 0.02 | 5.80 | 0.51 | 6 |  |
| Pugh et al 2017b [36] | 0.11 | 0.28 | 0.08 | -0.44 | 0.67 | 0.41 | 0.68 | 7.27 | -0.80 | 11 |  |
| Ryan et al 1996 [56] | 0.06 | 0.33 | 0.11 | -0.59 | 0.71 | 0.18 | 0.86 | 6.91 | -0.85 | 7 |  |
| van Nieuwenhoven et al 2004 [49] | -0.64 | 0.33 | 0.11 | -1.27 | 0.00 | -1.95 | 0.05 | 6.96 | -1.77 | 10 |  |
| Van Wijck et al 2012[11] | 0.00 | 0.30 | 0.09 | -0.60 | 0.60 | 0.00 | 1.00 | 7.11 | -0.94 | 9 |  |
| Van Wijck et al 2011 [12] | 0.23 | 0.35 | 0.13 | -0.46 | 0.93 | 0.66 | 0.51 | 6.75 | -0.61 | 6 |  |
| Zuhl et al 2014 [50] | 0.79 | 0.38 | 0.14 | 0.06 | 1.53 | 2.11 | 0.03 | 6.59 | 0.11 | 8 |  |
| Zuhl et al 2015 [51] | 3.40 | 0.98 | 0.95 | 1.49 | 5.31 | 3.48 | 0.00 | 2.93 | 2.26 | 7 |  |
| **TOTAL** | **0.70** | **0.21** | **0.04** | **0.30** | **1.11** | **3.40** | **<0.001** |  |  |  |  |

a, b, multiple studies by the same author published in the same year; (1),(2), studies that included multiple arms that were analysed separately due to different environmental or exercise conditions; ǂ, studies that have multiple arms with similar environmental or exercise conditions that are pooled for the meta-analysis to prevent overpowering

**Supplementary table 4: Summary of moderator variable analysis for feeding state, environment and timing of saccharide drinks by subgroup**

| **Moderator variable** | **p value** | **Comparison** |
| --- | --- | --- |
| *Fasted vs fed* |  |  |
| DS/MS ratio | 0.011 | Fasted (n=12) Hedges's g 1.15 (95%CI 0.53 – 1.79) |
|  |  | Fed (n=5) Hedges's g 0.20 (95% CI -0.18 – 0.58) |
| i-FABP | 0.949 | Fasted (n=9) Hedges's g 0.72 (95% CI 0.56 – 1.00) |
|  |  | Fed (n=14) Hedges’s g 0.81 (95% CI 0.51 – 1.10) |
| *Hot vs thermoneutral* |  |  |
| i-FABP | 0.007 | Hot (n=10) Hedges’s g 1.06 (95% CI 0.89 – 1.24) |
|  |  | Thermoneutral (n=16) Hedges’s g 0.66 (95 % CI 0.43 – 0.90) |
| *Time of drink* |  |  |
| DS/MS ratio | 0.005 | Before (n=4) Hedges’s g 0.05 (95% CI -0.34 – 0.45) |
|  |  | During (n=7) Hedges’s g 0.62 (95%CI 0.14 – 1.09) |
|  |  | After (n=6) Hedges’s g 2.01 (95% CI 0.82 – 3.33) |

**Supplementary table 5: Summary of the risk of bias for each study using the Cochrane risk of bias tool**

|  |  | **Selection bias** | | **Performance bias** | **Detection bias** | **Attrition bias** | **Reporting bias** | **Other bias** |
| --- | --- | --- | --- | --- | --- | --- | --- | --- |
|  |  | Random sequence generation | Allocation concealment | Blinding of participants and personnel | Blinding of outcome assessment | Incomplete outcome data | Selective reporting | Other sources of bias |
| **Authors** | **Year** | Decision | Decision | Decision | Decision | Decision | Decision | Decision |
| Ashton et al [55] | 2003 | Low risk | Low risk | Low risk | Low risk | Low risk | Unclear risk | Low risk |
| Buchman et al [52] | 1999a | Low risk | Low risk | Low risk | Low risk | Low risk | Unclear risk | Low risk |
| Buchman et al [7] | 1999b | Low risk | Low risk | Low risk | Low risk | Low risk | Unclear risk | Low risk |
| Davison et al [53] | 2016 | Low risk | Low risk | Low risk | Low risk | Low risk | Low risk | Low risk |
| JanssenDuijghuijsen et al [54] | 2017 | Low risk | Unclear risk | Low risk | Low risk | Low risk | Unclear risk | Low risk |
| JanssenDuijghuijsen et al [9] | 2016 | Low risk | Unclear risk | Low risk | Low risk | Low risk | Unclear risk | Low risk |
| Karhu et al [43] | 2017 | Low risk | Unclear risk | Low risk | Low risk | Low risk | Unclear risk | Low risk |
| Kartaram et al [44] | 2018 | Low risk | Low risk | Low risk | Low risk | Low risk | Unclear risk | Low risk |
| Lambert et al [48] | 2008 | Low risk | Low risk | Low risk | Low risk | Low risk | Unclear risk | Low risk |
| Lis et al [45] | 2015 | Low risk | Low risk | Low risk | Low risk | Low risk | Unclear risk | Low risk |
| March et al [47] | 2019 | Low risk | Unclear risk | Low risk | Low risk | Low risk | Low risk | Low risk |
| March et al [46] | 2017 | Low risk | Low risk | Low risk | Low risk | Low risk | Low risk | Low risk |
| Marchbank et al [28] | 2011 | Low risk | Low risk | Low risk | Low risk | Low risk | Low risk | Low risk |
| McKenna et al [33] | 2017 | Low risk | Low risk | Low risk | Low risk | Low risk | Unclear risk | Low risk |
| Morrison et al [34] | 2014 | Low risk | Low risk | Low risk | Low risk | Low risk | Unclear risk | Low risk |
| Pals et al [18]p | 1997 | Low risk | Unclear risk | Low risk | Low risk | Low risk | Unclear risk | Low risk |
| Pugh et al [35] | 2017a | Low risk | Unclear risk | Low risk | Low risk | Low risk | Unclear risk | Low risk |
| Pugh et al [36] | 2017b | Low risk | Low risk | Low risk | Low risk | Low risk | Unclear risk | Low risk |
| Ryan et al [56] | 1996 | Low risk | Low risk | Low risk | Low risk | Low risk | Unclear risk | Low risk |
| Sheahen et al [20] | 2018 | Low risk | Low risk | Low risk | Low risk | Low risk | Unclear risk | Low risk |
| Shing et al [57] | 2014 | Low risk | Low risk | Low risk | Low risk | Low risk | Unclear risk | Low risk |
| Snipe et al [39] | 2018a | Low risk | Unclear risk | Low risk | Low risk | Low risk | Unclear risk | Low risk |
| Snipe et al [38] | 2018b | Low risk | Unclear risk | Low risk | Low risk | Low risk | Unclear risk | Low risk |
| Snipe et al [59] | 2017 | Low risk | Unclear risk | Low risk | Low risk | Low risk | Unclear risk | Low risk |
| Snipe et al [40] | 2018c | Low risk | Low risk | Low risk | Low risk | Low risk | Unclear risk | Low risk |
| Szymanski et al [41] | 2018 | Low risk | Low risk | Low risk | Low risk | Low risk | Unclear risk | Low risk |
| van Nieuwenhoven et al [49] | 2004 | Low risk | Unclear risk | Low risk | Low risk | High risk | Unclear risk | Low risk |
| Van Wijck et al [11] | 2012 | Low risk | Unclear risk | Low risk | Low risk | Low risk | Unclear risk | Low risk |
| van Wijck et al [4] | 2013 | High risk | Unclear risk | Low risk | Low risk | Low risk | Unclear risk | Low risk |
| van Wijck et al [12] | 2011 | Low risk | Unclear risk | Low risk | Low risk | Low risk | Unclear risk | Low risk |
| van Wijck et al [42] | 2014 | Low risk | Low risk | Low risk | Low risk | Low risk | Unclear risk | Low risk |
| Yeh et al [58] | 2013 | Low risk | Unclear risk | Low risk | Low risk | Low risk | Unclear risk | Low risk |
| Zuhl et al [50] | 2014 | Low risk | Low risk | Low risk | Low risk | Low risk | Unclear risk | Low risk |
| Zuhl et al [51] | 2015 | Low risk | Low risk | Low risk | Low risk | Low risk | Unclear risk | Low risk |
