# Supplementary material for: The Effects of Exercise on Indirect Markers of Gut Damage and Permeability: A Systematic Review and Meta-analysis
Source: Sports Med. 2020 Nov 17;51(1):113–24. doi: 10.1007/s40279-020-01348-y (PMC7806566; doi:10.1007/s40279-020-01348-y)
Supplement: Supplementary file 2 — Supplementary file2 (DOCX 13 kb) [file 40279_2020_1348_MOESM2_ESM.docx]

**Supplementary material:**

Supplementary methods: search strategy for each database

PubMed

(("gut"[tiab] OR gastrointestinal[tiab] OR GI[tiab] OR "intestines"[MeSH Terms] OR "intestinal"[ tiab] OR "mucosal"[ tiab] OR splanchnic[tiab])

AND

("permeability"[MeSH Terms] OR "permeability"[tiab] OR leaky[tiab] OR hyperpermeability[tiab] OR "function"[ tiab] OR "dysfunction"[ tiab] OR "injury"[ tiab]))

AND

("exercise"[MeSH Terms] OR "exercise"[tiab] OR "training"[ tiab] OR endurance[tiab] OR "physical activity"[tiab])

NOT

("animals"[MeSH Terms] NOT "humans"[MeSH Terms])

MEDLINE, SPORTDiscus, and CINAHL via EBSCOhost

(((TI "gut" OR AB “gut”) OR (TI “gastrointestinal” OR AB “gastrointestinal”) OR (TI “GI” OR AB “GI”) OR (MH "intestines") OR (TI "intestinal" OR AB "intestinal") OR (TI "mucosal" OR AB “mucosal”) OR (TI “splanchnic” OR AB “splanchnic”))

AND ((MH "permeability") OR (TI "permeability" OR AB "permeability") OR (TI “leaky” AB “leaky”) OR (TI “hyperpermeability” OR AB “hyperpermeability”) OR (TI "function" OR AB “function”) OR (TI "dysfunction" OR AB “dysfunction”) OR (TI "injury" OR AB “injury”)))

AND ((MH "exercise") OR (TI "exercise" OR AB “exercise”) OR (TI "training" OR AB “training”) OR (TI “endurance” OR AB “endurance”) OR (TI "physical activity" OR AB “physical activity”))

NOT ((MH "animals") NOT (MH "humans"))

Cochrane library

#1 ("gut" or "gastrointestinal" or "GI" or "intestines" or "intestinal" or "mucosal" or "splanchnic"):ti,ab,kw

#2 ("Permeability" or "leaky" or "hyperpermeability" or "function" or "dysfunction" or "injury"):ti,ab,kw

#3 ("Exercise" or "training" or "endurance" or "physical activity"):ti,ab,kw

#4 MeSH descriptor: [Animals] explode all trees

#5 MeSH descriptor: [Humans] explode all trees

#6 (#1 and #2 and #3) not (#4 not #5)
